# Theoretical biological activities and docking studies of new derivatives of acyclovir for the treatment of coronavirus disease 2019

**DOI:** 10.25122/jml-2023-0335

**Published:** 2024-09

**Authors:** Muthanna Saadi Farhan

**Affiliations:** 1 Department of Pharmaceutical Chemistry, College of Pharmacy, University of Baghdad, Baghdad, Iraq

**Keywords:** acyclovir, antiviral agents, COVID-19, ligands, molecular docking simulation, one-click docking

## Abstract

Acyclovir is an established antiviral agent. The global emergence of the coronavirus disease 2019 (COVID-19) pandemic brought forth the necessity to investigate potential therapeutic attributes of existing drugs, including acyclovir, to combat this novel virus. The primary focus of this research was to assess the theoretical bioactivities of acyclovir derivatives and to evaluate their molecular docking capacities, thereby determining their prospective application in treating COVID-19. A set of 22 ligand molecules derived from acyclovir were carefully selected for this study. Using the one-click docking technique, these derivatives underwent molecular interactions with specific proteins sourced from the Protein Data Bank, identified by IDs 1R4L, 1S49, 1AJ6, and 1PVG. The molecular docking analysis revealed that acyclovir derivatives no. 3, 5, 8, and 14 displayed the highest docking scores and could be potential candidates as therapeutic agents against COVID-19 based on these scores. Further experimental validations are essential to establish their efficacy in clinical settings.

## INTRODUCTION

The global onset of coronavirus disease 2019 (COVID-19) was associated with an urgent need for effective treatment methods. Originating from a group of viruses known to cause illness in animals and humans, the severe acute respiratory syndrome coronavirus 2 (SARS-CoV-2) has proven itself to be exceptionally contagious and dangerous in humans, leading to the worldwide pandemic we know as COVID-19 [1,2]. In our endeavor to tackle this pandemic, scientists and researchers worldwide have been scrambling to find viable treatments by developing new drugs or repurposing existing ones [3,4].

Acyclovir, an antiviral medication, has been primarily used in the treatment of herpes simplex virus infections, varicella zoster (chickenpox), and other viral diseases. Given its established antiviral properties, there is an inherent interest in understanding its potential against newer and emerging viral strains, such as the novel coronavirus. Although the efficacy of acyclovir against herpes is well documented, its potential against SARS-CoV-2 remains an area of intrigue and active research [5,6].

The crux of this project revolves around enhancing the antiviral activity of acyclovir. Our goal is not only to use acyclovir in its native form, but also to explore new derivatives that might showcase enhanced or complementary properties against COVID-19. Repurposing drugs, such as acyclovir, is a strategic approach in drug discovery as it allows for the leveraging of pre-existing safety and pharmacokinetic data, potentially accelerating the path to clinical trials and eventual therapeutic use [7].

To efficiently screen the potential of acyclovir and its myriad derivatives, a holistic approach that involves computational biology is essential. Instead of the traditional methods of in vitro and in vivo screening, which can be both time-consuming and resource-intensive, in silico analyses offer a quicker preliminary assessment [8]. Molecular docking, a method that predicts the preferred orientation of one molecule to another when bound to each other to form a stable complex, is an invaluable tool in such endeavors. It can offer insights into the binding affinities of molecules, thereby hinting at their potential efficacy as drugs [9,10]. Our objective is twofold: first, to use computational tools for a preliminary screening of a series of 22 new prodrugs of acyclovir; second, to proceed with the synthesis of those derivatives that demonstrate superior binding affinity in these in silico studies. These new prodrugs could potentially lead to the development of a potent antiviral agent against COVID-19.

The significance of this research extends beyond the COVID-19 pandemic. The process of efficiently repurposing drugs through computational methods can set a precedent for tackling future viral outbreaks [11]. By quickly identifying potential drug candidates through in silico methods, the medical community can respond more promptly to emerging threats.

### The role of DNA gyrase and topoisomerase in SARS-CoV-2 infection

#### DNA gyrase

1. Viral structure and replication: SARS-CoV-2 is an RNA virus. RNA polymerase, not DNA gyrase, is the enzyme responsible for its replication. After creating new RNA copies, the virus assembles them into fresh viral particles using its RNA genome as a template [12,13].
2. Host cell impact: Upon infection, the virus enters host cells and hijacks the cellular machinery to replicate its RNA and produce viral proteins. This process is independent of the host cell’s DNA gyrase, as the virus does not integrate into the host DNA or require DNA manipulation for replication [14].

#### Potential connections

1. Secondary bacterial infections: Patients with COVID-19 may be more susceptible to secondary bacterial infections. In such cases, antibiotics that target DNA gyrase might be used; however, they are directed towards the co-infections rather than directly combating the virus [15].
2. Pharmacological research: Some research has explored whether drugs targeting enzymes like DNA gyrase could indirectly affect viral replication or the host’s immune response. However, such research is still in early stages and not specific to COVID-19 [16].

### Topoisomerase II

Topoisomerase II is an enzyme that has a crucial role in DNA replication, transcription, and chromosome segregation. However, it is not directly implicated in COVID-19, which is caused by an RNA virus. There may be indirect effects in which cellular stress or immune responses to the viral infection could impact enzymes like topoisomerase II, but these are not direct interactions with the virus itself [17].

### The role of RNA-dependent RNA polymerase (RdRp)

Also known as nsp12, RdRp has a pivotal role in the life cycle of SARS-CoV-2, with the following functions:

1. **Viral replication:** RdRp is essential for the replication of SARS-CoV-2. Given that SARS-CoV-2 is an RNA virus, it relies on RdRp to synthesize new RNA strands from its RNA genome. This is different from DNA viruses or cellular organisms that typically use DNA-dependent RNA polymerases [18].
2. **Transcription of viral genome:** RdRp not only replicates the viral genome but also transcribes the subgenomic RNAs that are used to produce viral proteins. These proteins are necessary for the assembly of new virus particles [19].
3. **Target for antiviral drugs:** Owing to its crucial role in viral replication, RdRp has been a major target of antiviral drugs such as remdesivir, an adenosine nucleotide analog, which are designed to inhibit RdRp. They work by incorporating into the newly synthesized RNA strand and causing premature termination of RNA synthesis [20]. The role of angiotensin-converting enzyme 2 in COVID-19 is also crucial in understanding the mechanism of infection and its widespread impact on various body systems [21].

In line with the purpose of this research, we will be using the molecular docking software 1-Click Docking to investigate the binding affinity and interaction of acyclovir derivatives. This software will be instrumental in mapping how these derivatives interact with the virus at a molecular level, providing insights that are critical for subsequent synthesis and potential clinical applications.

## Material and Methods

### Structure of ligands

The structure and IUPAC names of acyclovir and its derivatives are presented in Tables 1, 2, and 3.

**Table 1:**
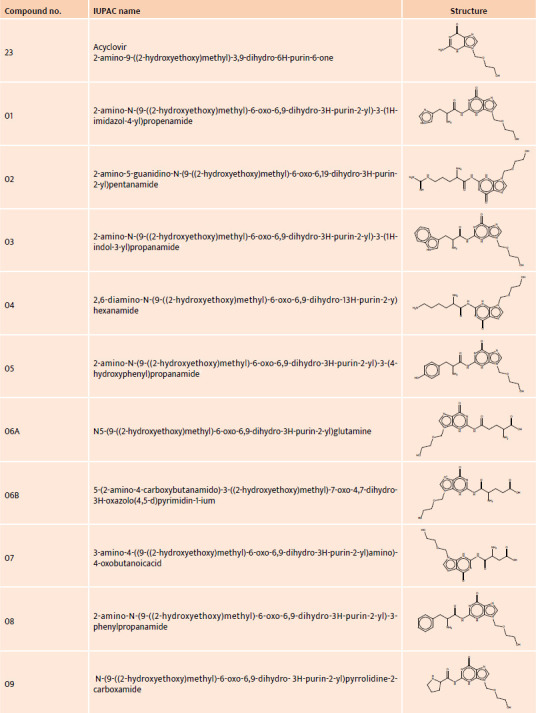
IUPAC name and structure of acyclovir (compound 23) and its derivatives (compounds 01–22)

**Table 2:**
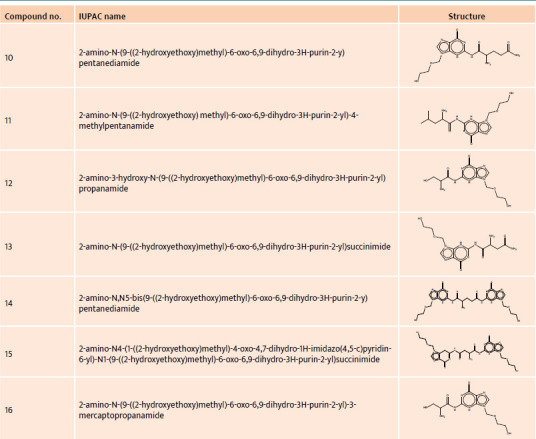
IUPAC name and structure of acyclovir derivatives no. 10–16

**Table 3:**
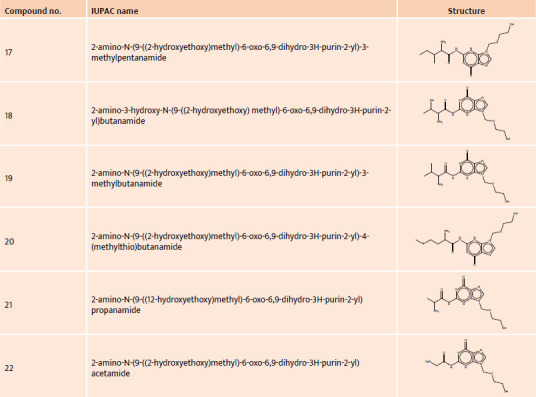
IUPAC name and structure of acyclovir derivatives no. 17–22

### Selection of protein structures

The target protein structures intended to be inhibited were identified and extracted from the Protein Data Bank (PDB) for the purpose of molecular docking research [22]. The selected proteins and their respective PDB IDs are as follows:

- angiotensin-converting enzyme (PDB ID: 1R4L);
- RdRp (PDB ID: 1S49);
- DNA gyrase subunit B (PDB ID: 1AJ6);
- DNA topoisomerase 2 (PDB ID: 1PVG).

### Molecular docking simulations

To ascertain the optimal position for protein–ligand binding interactions, docking simulations were performed using the 1-Click Docking online platform (mcule.com/apps/1-click-docking/). This provided insights into the binding affinity and interaction of the ligands with the selected proteins, facilitating the identification of promising drug candidates.

### Drawing of compound structures

The two-dimensional molecular structures of the compounds under investigation were sketched using ChemDraw software v.19.0 (PerkinElmer). This helped in visual representation and understanding of the structural nuances of the molecules.

### Docking software

All molecular docking operations were conducted on a Windows 10 operating system using the docking program specified above. The software allowed for rigorous computational simulations to determine how the ligands interact with the target proteins, offering predictions about potential drug efficacies.

## RESULTS

During the molecular docking studies we observed that new therapeutic agents presented notable theoretical docking scores on the four key receptors that we investigated (Table 4). These scores were compared with acyclovir to gauge the potential of these derivatives. Compounds no. 3, 5, 8, and 14 emerged as particularly promising candidates for the treatment of COVID-19 based on their docking scores.

**Table 4:** Docking results of acyclovir and its derivatives

	Docking scores			
Compound no.	Angiotensin-converting enzyme (1R4L)	RdRp (1S49)	DNA gyrase subunit B (1AJ6)	DNA topoisomerase 2 (1PVG)
23	−6.7	−5.7	−6.6	−6.8
01	−7.6	−6.6	−6.5	−8.6
02	−7.7	−6.8	−7.0	−9.0
03	−8.8	−6.9	−8.5	−10.5
04	−7.1	−5.8	−6.5	−8.6
05	−8.8	−7.6	−7.8	−9.0
06A	−7.4	−5.9	−5.8	−8.8
06B	−7.7	−6.4	−7.0	−8.9
07	−7.5	−6.3	−6.8	−8.8
08	−8.1	−7.0	−7.6	−9.2
09	−8.2	−6.8	−6.4	−9.6
10	−7.1	−6.0	−5.8	−8.5
11	−8.1	−6.5	−6.9	−8.8
12	−7.4	−6.1	−6.5	−7.9
13	−7.8	−6.1	−5.9	−8.5
14	−9.4	−6.3	−7.5	−9.1
15	−8.8	−6.8	−7.0	−8.9
16	−7.2	−6.0	−5.9	−7.8
17	−7.8	−6.4	−6.6	−8.7
18	−7.6	−5.9	−6.4	−8.4
19	−7.6	−6.6	−7.1	−8.7
20	−7.7	−6.2	−5.7	−7.9
21	−7.5	−5.6	−7.0	−8.2
22	−7.2	−6.1	−6.4	−7.9

**Figure 1:**
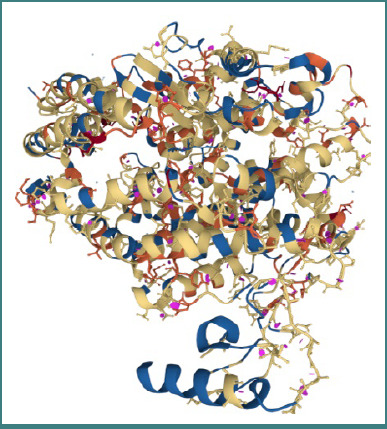
Angiotensin-converting enzyme (1R4L). Source: Protein Data Bank.

**Figure 2:**
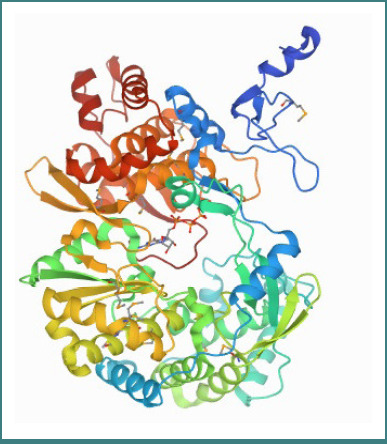
RdRp (1S49). Source: Protein Data Bank.

**Figure 3:**
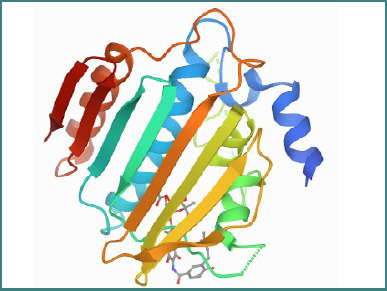
DNA gyrase subunit B (1AJ6). Source: Protein Data Bank.

**Figure 4:**
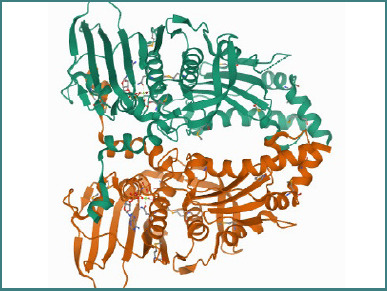
DNA topoisomerase 2 (1PVG). Source: Protein Data Bank.

For angiotensin-converting enzyme (1R4L), all acyclovir derivatives showed a lower energy score than acyclovir itself, indicating a stronger binding affinity. Derivatives with the best results were no. 14, 15, 3, and 5 (Figure 1). For RdRp (1S49), some acyclovir derivatives showed a lower energy score than acyclovir itself. Derivatives with the best results were no. 5 and 8 (Figure 2). For DNA gyrase subunit B (1AJ6), most acyclovir derivatives showed a lower energy score than acyclovir itself. Derivatives with the best results were no. 3, 5, 8 and 14 (Figure 3). For DNA topoisomerase 2 (1PVG), all acyclovir derivatives showed a lower energy score than acyclovir itself. Derivatives with the best results were no. 3, 9, 8, 14, 2 and 5 (Figure 4).

## Discussion

The quest for an effective therapeutic agent against SARS-CoV-2 has led to the exploration of a plethora of potential drug candidates [23–25]. In this study, we focused on acyclovir, a well-established antiviral medication, and its derivatives [26–28]. The decision to explore acyclovir is underpinned by its longstanding use in the treatment of herpes simplex virus infections, suggesting that its mechanism of action might offer some efficacy against SARS-CoV-2 [29].

Molecular docking, an in silico analytical tool, serves as a preliminary step to screen drug candidates by analyzing their binding affinity to specific target proteins. The lower energy scores observed for many of the acyclovir derivatives compared to acyclovir itself indicate stronger interactions and potentially increased efficacy. These scores essentially provide a metric to determine how well a molecule might bind to and potentially inhibit the function of the targeted protein [30,31].

Several receptors were targeted in our study. Among them, angiotensin-converting enzyme (1R4L) is of particular interest, given its role in facilitating the entry of the virus into host cells. The fact that all acyclovir derivatives exhibited lower energy scores than acyclovir for this receptor is promising, as it implies these derivatives may be more effective at preventing viral entry.

For RdRp (1S49), an essential enzyme for viral replication, only certain derivatives outperformed acyclovir. This enzyme has been the focus of many antiviral strategies, also being targeted with remdesivir, an approved treatment for COVID-19. Any compound that demonstrates a high binding affinity to this enzyme could, in theory, hamper the replication of the virus.

The results concerning DNA gyrase subunit B (1AJ6) and DNA topoisomerase 2 (1PVG) are also enlightening [31]. These enzymes have roles in DNA replication, repair, and recombination. Although SARS-CoV-2 is an RNA virus, disrupting host DNA processes could still potentially impact the viral life cycle or the host’s response. The primary intersection between DNA gyrase and COVID-19 may occur in the context of secondary bacterial infections in patients with COVID-19 or in broader pharmacological research. Understanding these distinctions is vital for effective treatment strategies and for ongoing research in antiviral therapies. The standout performance of specific acyclovir derivatives (notably no. 3, 5, 8, and 14) may offer avenues for further exploration.

It is essential to emphasize that while molecular docking provides valuable preliminary insights, it is not definitive. In vivo and in vitro studies are imperative to validate these findings, considering various factors such as drug metabolism, potential side effects, and actual inhibitory concentration levels in a biological setting.

Furthermore, while the derivatives show promise, the synthesis of these derivatives on a large scale and their subsequent pharmacokinetic and pharmacodynamic profiles would need to be assessed. Also, while acyclovir has a known safety profile, its derivatives might behave differently in human systems.

## Conclusion

This study used molecular docking to evaluate the potential efficacy of acyclovir and its derivatives against specific targets related to COVID-19. Several acyclovir derivatives, notably compounds no. 3, 5, 8, and 14, demonstrated promising binding affinities, surpassing acyclovir itself. These findings suggest that acyclovir derivatives could be potential candidates for the treatment of COVID-19, warranting further in-depth in vivo and in vitro investigations.

## Supplementary Material



## Data Availability

Data analyzed in this article are available in the Zenodo repository at https://zenodo.org/records/8367616, with the DOI 10.5281/zenodo.8367616. All data are available under Creative Commons Attribution 4.0 International.

## References

[1] Yilmazkuday H (2022). Coronavirus disease 2019 and the global economy. Transport Policy.

[2] Erdem I, Ardic E, Turker E, Kardan ME, Demirkapu MJ (2022). Comparison of antibiotic use in the COVID-19 pandemic with the pre-pandemic period in a university hospital. Archives of Medical Science: AMS.

[3] Zhang L, Guo H (2020). Biomarkers of COVID-19 and technologies to combat SARS-CoV-2. Advances in Biomarker Sciences and Technology.

[4] Salman OD, Ibrahim SK, Rashid RG, Al-Hussaniy HA (2023). The role of laboratory test biomarkers in diagnosis, risk assessment, and monitoring of COVID-19 patients. Journal of Clinical Trials and Experimental Investigations.

[5] Al-juhaishi AM, Aziz ND (2022). Safety and efficacy of antiviral drugs against covid-19 infection: an updated systemic review. Medical and Pharmaceutical Journal.

[6] Awad M, Al-Hussaniy HA, Alburghaif AH, Tawfeeq KT (2022). The role of COVID-19 in myopathy: incidence, causes, treatment, and prevention. Journal of Medicine and Life.

[7] Mohandoss S, Sukanya R, Ganesan S, Alkallas FH, Trabelsi AB, Kusmartsev FV, Velu KS, Stalin T, Lo HM, Lee YR (2022). SARS-CoV-2 main protease (3CLpro) interaction with acyclovir antiviral drug/methyl-β-cyclodextrin complex: Physiochemical characterization and molecular docking. Journal of Molecular Liquids.

[8] Singh MB, Jain P, Tomar J, Kumar V, Bahadur I, Arya DK, Singh P (2022). An In Silico investigation for acyclovir and its derivatives to fight the COVID-19: Molecular docking, DFT calculations, ADME and td-Molecular dynamics simulations. Journal of the Indian Chemical Society.

[9] Khan T, Raza S (2023). Exploration of Computational Aids for Effective Drug Designing and Management of Viral Diseases: A Comprehensive Review. Curr Top Med Chem.

[10] Othman Makki Sagheer, Manar Serhan Ahmed, Mazen Mohammed Jwaid, Zahraa alkhafaje, Hany akeel Al-hussaniy, Zahraa S. Al-Tameemi (2023). The development of molecular docking and molecular dynamics and their application in the field of chemistry and computer simulation. Journal of medical pharmaceutical and allied sciences.

[11] Heidari A, Navimipour NJ, Unal M, Toumaj S (2022). The COVID-19 epidemic analysis and diagnosis using deep learning: A systematic literature review and future directions. Computers in Biology and Medicine.

[12] Cardoso-Ortiz J, Leyva-Ramos S, Baines KM, Gómez-Durán CF, Hernández-López H, Palacios-Can FJ, Valcarcel-Gamiño JA, Leyva-Peralta MA, Razo-Hernández RS (2023). Novel ciprofloxacin and norfloxacin-tetrazole hybrids as potential antibacterial and antiviral agents: Targeting S. aureus topoisomerase and SARS-CoV-2-MPro. Journal of Molecular Structure.

[13] Navya VB, Hosur MV (2021). A computational study on hydroxychloroquine binding to target proteins related to SARS-COV-2 infection. Informatics in Medicine Unlocked.

[14] Alaaeldin R, Mustafa M, Abuo-Rahma GE, Fathy M (2022). In vitro inhibition and molecular docking of a new ciprofloxacin-chalcone against SARS-CoV-2 main protease. Fundamental & Clinical Pharmacology.

[15] Al-hussaniy H, Kadhim Z (2022). Methicillin-Resistant Staphylococcus aureus and New Delhi Metallo beta-lactamases-types of antibiotic resistance, methods of prevention. Medical and Pharmaceutical Journal.

[16] Hasan MR, Chowdhury SM, Aziz MA, Shahriar A, Ahmed H, Khan MA, Mahmud S, Emran TB (2021). In silico analysis of ciprofloxacin analogs as inhibitors of DNA gyrase of Staphylococcus aureus. Informatics in Medicine Unlocked.

[17] Lovetrue B (2020). The AI-discovered aetiology of COVID-19 and rationale of the irinotecan+ etoposide combination therapy for critically ill COVID-19 patients. Medical hypotheses.

[18] Hillen HS (2021). Structure and function of SARS-CoV-2 polymerase. Current opinion in virology.

[19] Naydenova K, Muir KW, Wu LF, Zhang Z, Coscia F, Peet MJ, Castro-Hartmann P, Qian P, Sader K, Dent K, Kimanius D (2021). Structure of the SARS-CoV-2 RNA-dependent RNA polymerase in the presence of favipiravir-RTP. Proceedings of the National Academy of Sciences.

[20] Khattab ES, Ragab A, Abol-Ftouh MA, Elhenawy AA (2022). Therapeutic strategies for Covid-19 based on molecular docking and dynamic studies to the ACE-2 receptors, Furin, and viral spike proteins. Journal of Biomolecular Structure and Dynamics.

[21] Akeel Al-Hussaniy H. S, Al-tameemi Z, AL-Zobaidy MJ (2022). In silico comparison between the mutated and wild-type androgen receptors and their influence on the selection of optimum androgenic receptor blockers for the treatment of prostate cancer. F1000Research.

[22] Upreti S, Prusty JS, Pandey SC, Kumar A, Samant M (2021). Identification of novel inhibitors of angiotensin-converting enzyme 2 (ACE-2) receptor from Urtica dioica to combat coronavirus disease 2019 (COVID-19). Molecular Diversity.

[23] Salih TM (2022). A Comparative Study for the Accuracy of Three Molecular Docking Programs Using HIV-1 Protease Inhibitors as a Model. Iraqi Journal of Pharmaceutical Sciences.

[24] Alwan SM, Al-Mudhafar MM, Abdul-Wahab AH (2017). Synthesis, Preliminary Antimicrobial Evaluation, and Molecular Docking of new Schiff bases of Ceftizoxime. Journal of Pharmaceutical Sciences and Research.

[25] Maurya VK, Kumar S, Bhatt ML, Saxena SK (2020). Therapeutic Development and Drugs for the Treatment of COVID-19. Coronavirus Disease 2019 (COVID-19) Epidemiology, Pathogenesis, Diagnosis, and Therapeutics.

[26] Stoian M, Procopiescu B, Șeitan S, Scarlat G (2023). Post-COVID-19 syndrome: Insights into a novel post-infectious systemic disorder. Journal of medicine and life.

[27] Beshna E, Ashour RA, Layas NA, Eldawi NS (2023). Pharmacovigilance knowledge, attitudes, and practices of pharmacists in Zawia (Libya). Medical and Pharmaceutical Journal.

[28] Alkharsah KR, Wanni NH, Alsaffar R, Al Dossary R, Obeid OE, Al Qahtani N, Hunasemarada BC, El-Badry AA (2022). Prevalence of herpes simplex virus types 1 and 2 antibodies among individuals screened in a tertiary hospital in the Eastern province of Saudi Arabia. Journal of Medicine and Life.

[29] Subbaiyan A, Ravichandran K, Singh SV (2020). In silico Molecular Docking Analysis Targeting SARS-CoV-2 Spike Protein and Selected Herbal Constituents. J Pure Appl Microbiol.

[30] Kondaka K, Gabriel I (2022). Targeting DNA Topoisomerase II in Antifungal Chemotherapy. Molecules.

[31] Roney M, Issahaku AR, Forid MS, Huq AM, Soliman ME, Mohd Aluwi MF, Tajuddin SN (2023). In silico evaluation of usnic acid derivatives to discover potential antibacterial drugs against DNA gyrase B and DNA topoisomerase IV. Journal of Biomolecular Structure and Dynamics.

