# Supplementary material for: Theoretical biological activities and docking studies of new derivatives of acyclovir for the treatment of coronavirus disease 2019
Source: J Med Life. 2024 Sep;17(9):840–7. doi: 10.25122/jml-2023-0335 (PMC11611060; doi:10.25122/jml-2023-0335)
Supplement: Supplementary file 1 [file JMedLife-17-840-s001.pdf]

Supplementary data. Docking results visualized using the 1-Click Docking platform

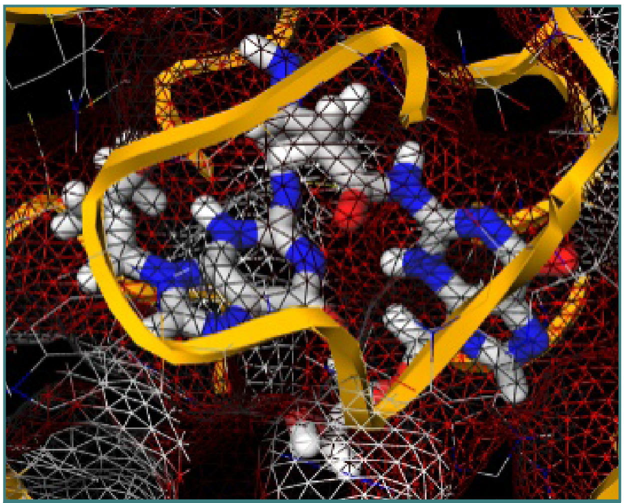

Supplementary Figure 1. Best pose of compound no. 14 on 1R4L receptor

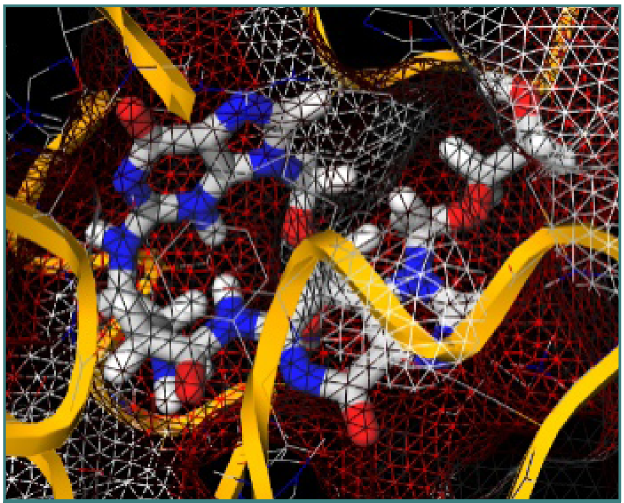

Supplementary Figure 2. Best pose of compound no. 15 with 1R4L receptor

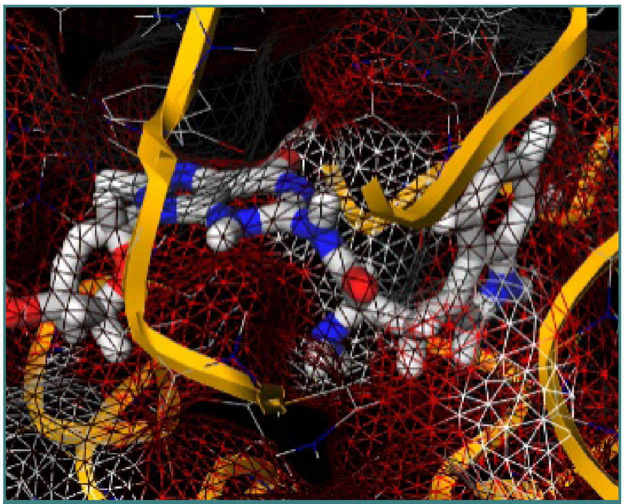

Supplementary Figure 3. Best pose of compound no. 3 with 1R4L receptor

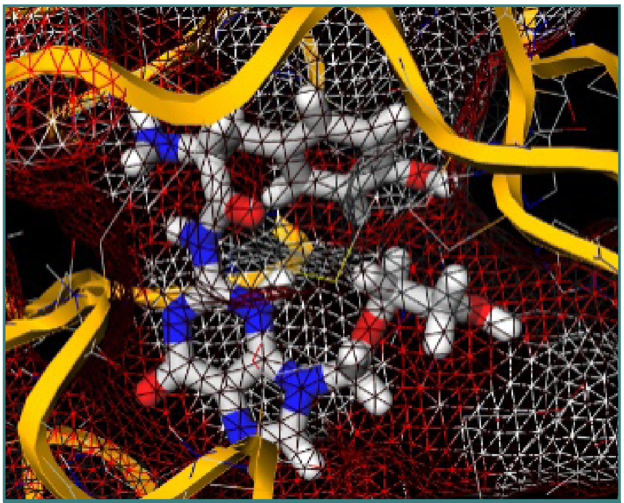

Supplementary Figure 4. Best pose of compound no. 5 with 1R4L receptor

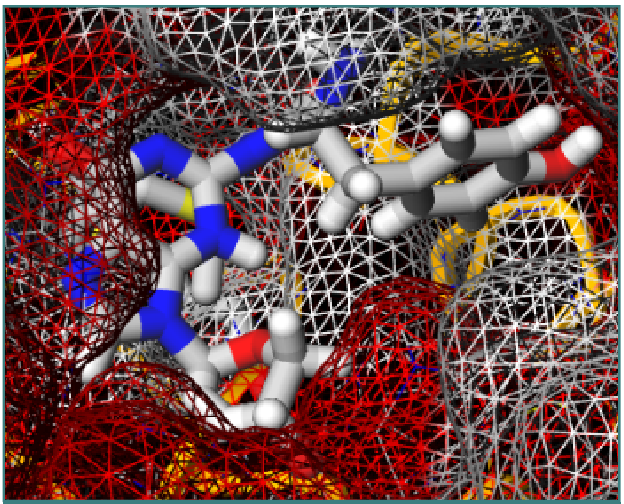

Supplementary Figure 5. Best pose of compound no. 5 on 1S49 receptor

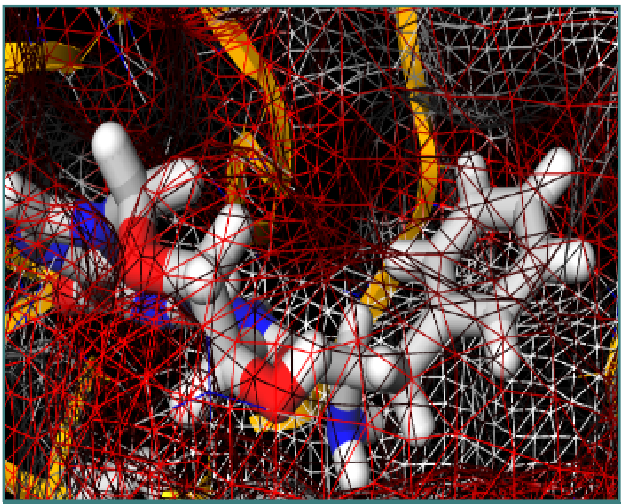

Supplementary Figure 6. Best pose of compound no. 8 on 1S49 receptor

© 2024 by the authors. This article is an open access article distributed under the terms and conditions of the Creative Commons Attribution (CC BY 4.0) license.

Supplementary data. Docking results visualized using the 1-Click Docking platform

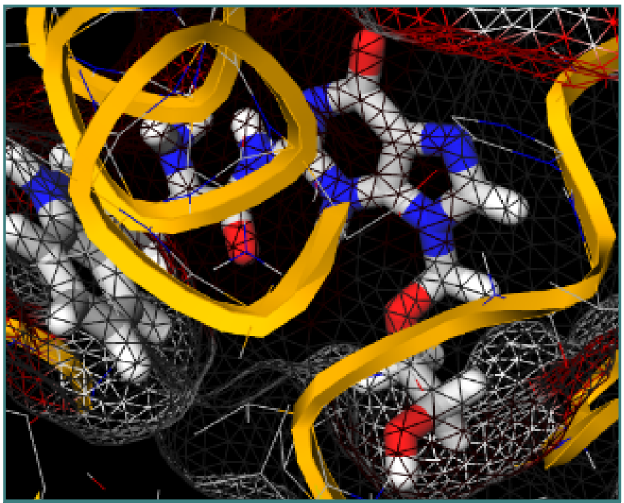
Supplementary Figure 7. Best pose of compound no. 3 on 1AJ6 receptor

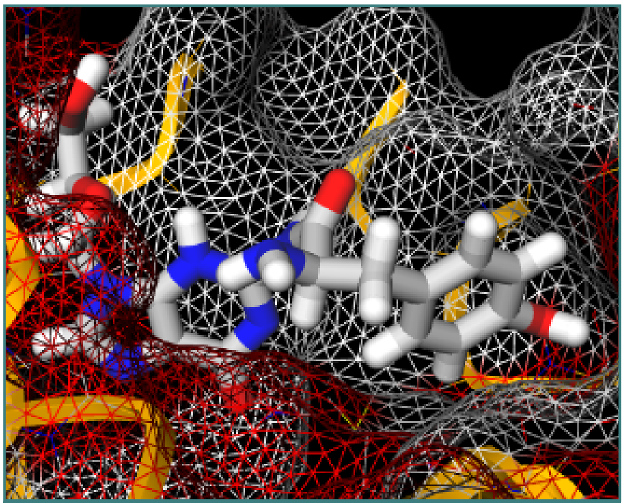
Supplementary Figure 8. Best pose of compound no. 5 on 1AJ6 receptor

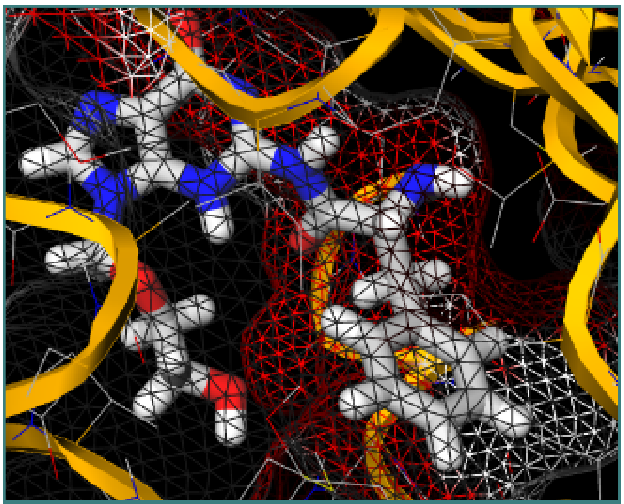
Supplementary Figure 9. Best pose of compound no. 8 on 1AJ6 receptor

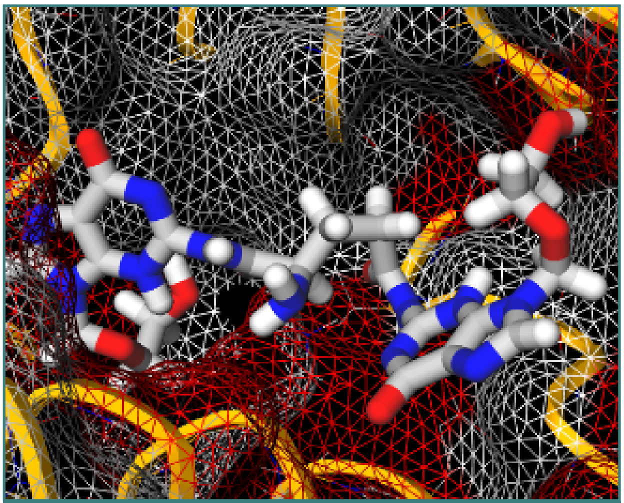
Supplementary Figure 10. Best pose of compound no. 14 on 1AJ6 receptor

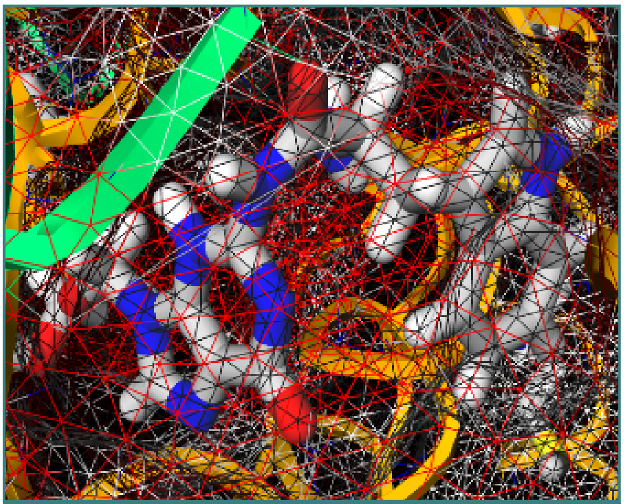
Supplementary Figure 11. Best pose of compound no. 3 on 1PVG receptor

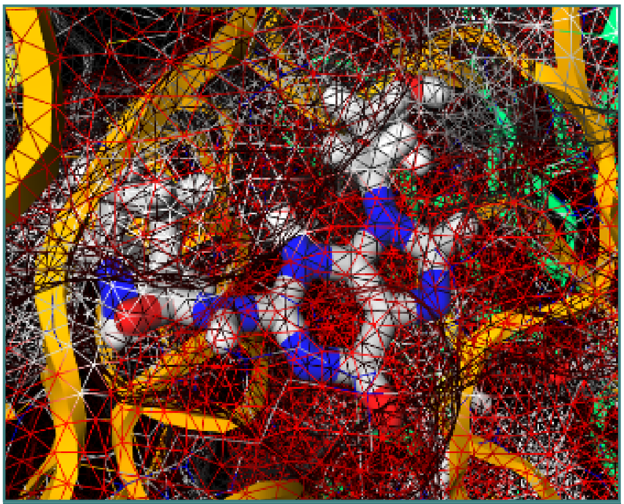
Supplementary Figure 12. Best pose of compound no. 9 on 1PVG receptor

© 2024 by the authors. This article is an open access article distributed under the terms and conditions of the Creative Commons Attribution (CC BY 4.0) license.

Supplementary data. Docking results visualized using the 1-Click Docking platform

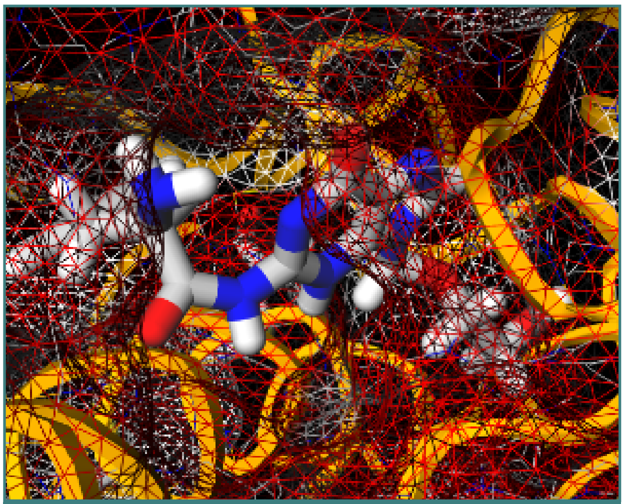

Supplementary Figure 13. Best pose of compound no. 8 on 1PVG receptor

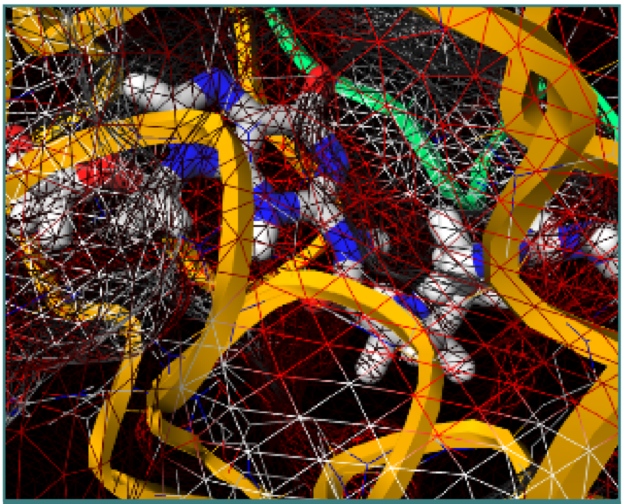

Supplementary Figure 15. Best pose of compound no. 2 on 1PVG receptor

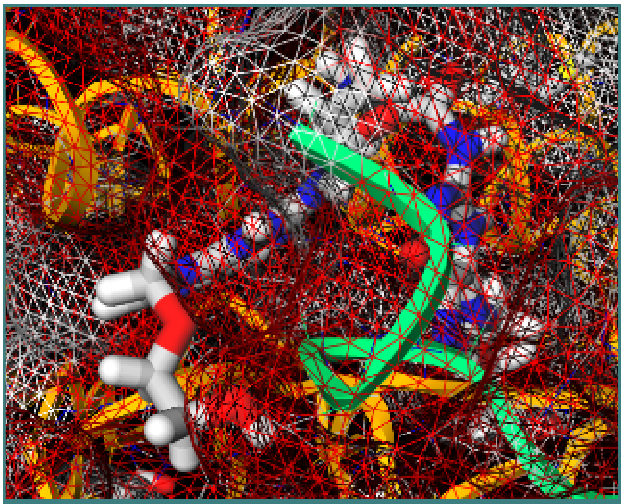

Supplementary Figure 14. Best pose of compound no. 14 on 1PVG receptor

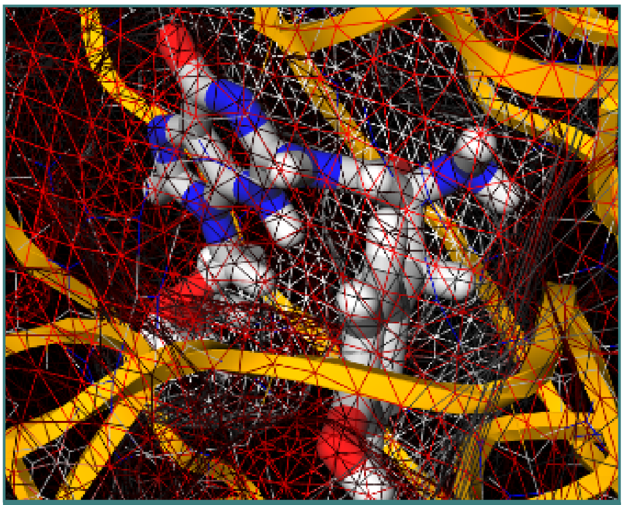

Supplementary Figure 16. Best pose of compound no. 5 on 1PVG receptor

© 2024 by the authors. This article is an open access article distributed under the terms and conditions of the Creative Commons Attribution (CC BY 4.0) license.
